# Three‐Year Follow‐Up of Clinical, Radiographic, and Patient‐Reported Outcomes for Zirconia and Titanium Implants in the Posterior Region: A Randomized Controlled Trial

**DOI:** 10.1111/clr.70176

**Published:** 2026-10-11

**Authors:** Anina N. Zuercher, Ronald E. Jung, Stefan P. Bienz, Stephanie A. Wenk, Daniel S. Thoma, Marc Balmer

**Affiliations:** ^1^ Clinic of Reconstructive Dentistry, Center for Dental Medicine University of Zurich Zurich Switzerland

**Keywords:** biocompatible materials, ceramics, dental implants, osseointegration, zirconia implants, zirconium oxide

## Abstract

**Objective:**

To compare the 3‐year clinical, radiographic, esthetic, and patient‐reported outcomes of zirconia (Zr) and titanium (Ti) implants placed in posterior sites in a randomized, controlled within‐subject design.

**Materials and Methods:**

Forty‐two patients each received one Zr and one Ti implant. Clinical and radiographic outcomes were evaluated at baseline, 1, and 3 years after crown delivery. Marginal bone levels (MBL), peri‐implant soft‐tissue parameters, technical complications, esthetic outcomes (Δ*E* values, VAS scores), and patient‐reported measures were assessed. Thirty‐nine patients (37 Zr; 39 Ti implants) were available at 3 years.

**Results:**

The 3‐year survival rate was 100% for Ti and 94.9% for Zr implants. MBL remained stable after the initial remodeling phase, with no clinically relevant differences between materials. Peri‐implant soft‐tissue conditions (BOP, plaque presence, PD, KT) were similar for Zr and Ti. Two zirconia implants were lost during the observation period. Peri‐implantitis was diagnosed in 7.9% of the implants, affecting three titanium and three zirconia implants. Technical complications differed between groups: chipping occurred significantly more often at Zr implant‐supported crowns. Ti implants showed two screw‐loosening events, while Zr implants showed two cases of decementation. Esthetic and patient‐reported outcomes were comparable, with low perceived mucosal color differences and a slight preference for Zr sites, if detectable.

**Conclusion:**

Zr and Ti implants showed similar peri‐implant health, marginal bone stability, esthetic outcomes, and patient satisfaction after 3 years. Zr implants, however, demonstrated a slightly lower survival rate and more chipping.

**Trial Registration:**

The trial is registered in the German Clinical Trials Register (DRKS) under the ID DRKS00008591

## 1 Introduction

While titanium implants remain the best‐documented option with long‐term clinical data (Buser et al. 2012; Jung et al. 2021, 2013), zirconia implants have become an important alternative in implant dentistry. Beyond the increasing demand for metal‐free treatment concepts among patients, the tooth‐colored appearance and favorable biocompatibility of zirconia as a dental material may offer specific advantages over titanium in selected clinical situations (Balmer et al. 2020; Gahlert et al. 2016; Kohal et al. 2016). However, despite offering an alternative that may better address certain patient preferences, it remains unclear to what extent these materials differences translate into clinically relevant outcomes. In particular, data comparing zirconia and titanium implants with respect to peri‐implant health, technical complications, and patient satisfaction over time are still limited (Balmer, Payer, et al. 2026).

Peri‐implant health is closely linked to the stability of the surrounding hard and soft tissues, with marginal bone levels remaining a key indicator of mid‐ to long‐term implant stability (Albrektsson et al. 1986; Berglundh et al. 2018). Titanium implants reliably demonstrate low bone remodeling after the first year of loading, with stable marginal bone levels documented over 3 to even 20 years of follow‐up (Buser et al. 2012; Jung et al. 2021). Several prospective clinical studies of zirconia implants—although based on smaller cohorts and shorter follow‐up periods—report outcomes up to 5–10 years, demonstrating high survival rates with minimal marginal bone loss (Balmer, Payer, et al. 2026; Kohal et al. 2020; Roehling et al. 2026). Taken together, the data suggests that zirconia implants may perform comparably to titanium in terms of maintaining stable marginal bone levels over the mid‐term.

Another important aspect in the mid‐term evaluation of implant performance is the condition and stability of the peri‐implant soft tissues. Clinical findings have shown reduced plaque accumulation and lower bleeding tendencies around zirconia implants under experimentally induced mucositis conditions (Bienz et al. 2021). Clinical outcomes from the same Cohort 1 year after loading further suggest that zirconia and titanium implants demonstrate similar clinical soft‐tissue parameters (Zuercher et al. 2024). These observations are consistent with systematic reviews reporting no clinically relevant differences in clinical outcomes between zirconia and titanium implants (Padhye et al. 2023).

Esthetic outcomes, the color of soft tissues, technical complications, and patient satisfaction are additional key aspects of implant performance. The tooth‐colored appearance of zirconia has been associated with reduced mucosal discoloration, particularly in patients with thin, soft‐tissue biotypes (Jung et al. 2007; Thoma et al. 2016). Technical complications have been documented for both materials; titanium implants and their implant‐retained reconstructions are more frequently associated with screw loosening (Jung et al. 2012; Sailer et al. 2022), whereas zirconia implant crowns—especially earlier one‐piece designs—have shown a higher incidence of abutment or implant fractures (Hashim et al. 2016; Roehling et al. 2018). Patient satisfaction is closely linked to both esthetic appearance and technical reliability (Canallatos et al. 2020). Although patients generally report high satisfaction with both zirconia and titanium implant crowns (Zuercher et al. 2024), comparative mid‐term data assessing differences between the two systems remain limited. Furthermore, mid‐term esthetic outcomes and patient‐reported measures are still scarce, and direct comparative studies between zirconia and titanium implants beyond the first year are largely lacking (Balmer, Spies, et al. 2026; Mohseni et al. 2023).

Therefore, the present study aims to report the 3‐year follow‐up data on the clinical, radiographic, esthetic, and patient‐reported outcomes of zirconia and titanium implants placed in posterior regions.

## 2 Materials and Methods

### 2.1 Study Design and Population

This randomized controlled trial evaluated the mid‐term implant performance of zirconia and titanium implants after 3 years of functional loading. The study follows the same cohort and protocol described in earlier publications from this research group (Bienz et al. 2021; Zuercher et al. 2024). The first publication reported clinical and histological data after experimentally induced mucositis at 4 months following implant placement, and the second presented the 1‐year outcomes after loading. The primary endpoint of the project was the number of inflammatory cells at the level of the junctional epithelium after the experimental mucositis phase. Implant survival, clinical, radiographic, technical, esthetic, and patient‐reported outcomes at the 1‐, 3‐, and 5‐year follow‐ups were defined as secondary endpoints in the study protocol. This publication reports the 3‐year follow‐up results.

The parental study received approval from the local ethics committee (KEK_ZH‐No. 2015‐0215) and was registered in the German Clinical Trials Registry (DRKS00008591; 2015‐06‐18). This randomized controlled trial was conducted and reported in accordance with the CONSORT 2025 guidelines. Between August 2015 and February 2019, patients were continuously recruited and enrolled by the treating investigators at the Clinic of Reconstructive Dentistry, Center for Dental Medicine, University of Zurich, Switzerland. All participants provided written informed consent, and the trial was conducted in accordance with the Declaration of Helsinki.

### 2.2 Inclusion/Exclusion Criteria

A detailed outline of the inclusion and exclusion criteria has been described in detail in the previous publications (Bienz et al. 2021; Zuercher et al. 2024).

The study included male and female patients showing the following criteria:

- Two adjacent missing teeth in position of the premolars and molars in the maxilla or mandible
- At least one adjacent tooth to edentulous site
- Aged between 18 and 80 years
- No medical conditions preventing implant treatment
- At least 10 mm of vertical bone height in the mandible or 6 mm of vertical bone height in the maxilla
- A minimum of 2 mm keratinized mucosa

Exclusion criteria included inadequate oral hygiene (full‐mouth plaque control record > 30%), insufficient inflammation control (full‐mouth bleeding on probing score > 25%), active periodontal diseases, smoking more than 15 cigarettes per day, and pregnant or lactating women.

### 2.3 Clinical Procedure

Implant placement and the early clinical procedures have been described in detail in previous publications on this cohort (Bienz et al. 2021; Zuercher et al. 2024). In brief, each patient received one zirconia and one titanium implant in a split‐mouth design, meaning that both implant types were placed in the same patient to minimize inter‐individual variability. The allocation of zirconia and titanium implants to the mesial or distal position was performed using a computer‐generated randomization sequence (*n* = 20 per allocation sequence; www.random.org), which was generated by a study coordinator not involved in patient treatment. The allocation was concealed in sealed envelopes and implemented at the time of surgery by the treating clinician. Due to the nature of the intervention and the distinct clinical and radiographic appearances of the two implant systems, blinding of the surgeon and outcome examiners was not feasible; however, statistical data analysis was performed using coded group allocations and was therefore blinded.

Zirconia implants consisted of a one‐piece monotype design (PURE Ceramic Implant Ø4.1 mm, ZLA, Institut Straumann AG) made from yttrium‐stabilized zirconium dioxide. Titanium implants were two‐piece systems with a separate abutment (Standard Plus Implant Ø4.1 mm, SLA, Institut Straumann AG) made from commercially pure titanium. Guided bone regeneration (GBR) was done for any buccal defects, fenestrations, or contour augmentations (BioOss and/or BioOss Collagen with BioGide membrane, Geistlich Pharma AG, Wolhusen, Switzerland). All implants were placed according to standard protocols and allowed to heal transmucosally for 4–6 months. Patients received oral hygiene instructions and rinsed twice daily with 0.2% chlorhexidine (Kantonsapotheke Zürich, Zurich, Switzerland) until the wound check. Patients undergoing GBR received Amoxicillin Sandoz (750 mg; Sandoz Pharmaceuticals AG, Rotkreuz, Switzerland) twice daily for 5–7 days. Mefenacid (500 mg; Streuli Pharma AG, Uznach, Switzerland) was prescribed up to three times daily as needed for pain relief.

Following completion of the healing phase, implant cleaning was discontinued for 3 weeks in half of the patients (non‐cleaning group), whereas the remaining patients maintained their regular oral hygiene procedures (cleaning group), thereby allowing assessment of the biological response to experimentally induced mucositis. At the end of the experimental phase, a peri‐implant soft tissue biopsy was harvested from the inter‐implant soft tissue for histological analysis, as previously reported (Bienz et al.). After the healing period, both implant types were restored with monolithic zirconia crowns. For zirconia implants, crowns were directly cemented (Panavia 21, Kuraray, Osaka Japan) onto the monotype implant, whereas titanium implants received crowns cemented extraorally (Panavia 21) onto a titanium base and subsequently screw‐retained. Baseline examinations were performed 7–10 days after crown delivery, followed by a structured maintenance program with follow‐up visits up to 3 years (Figure 1).

**FIGURE 1:**
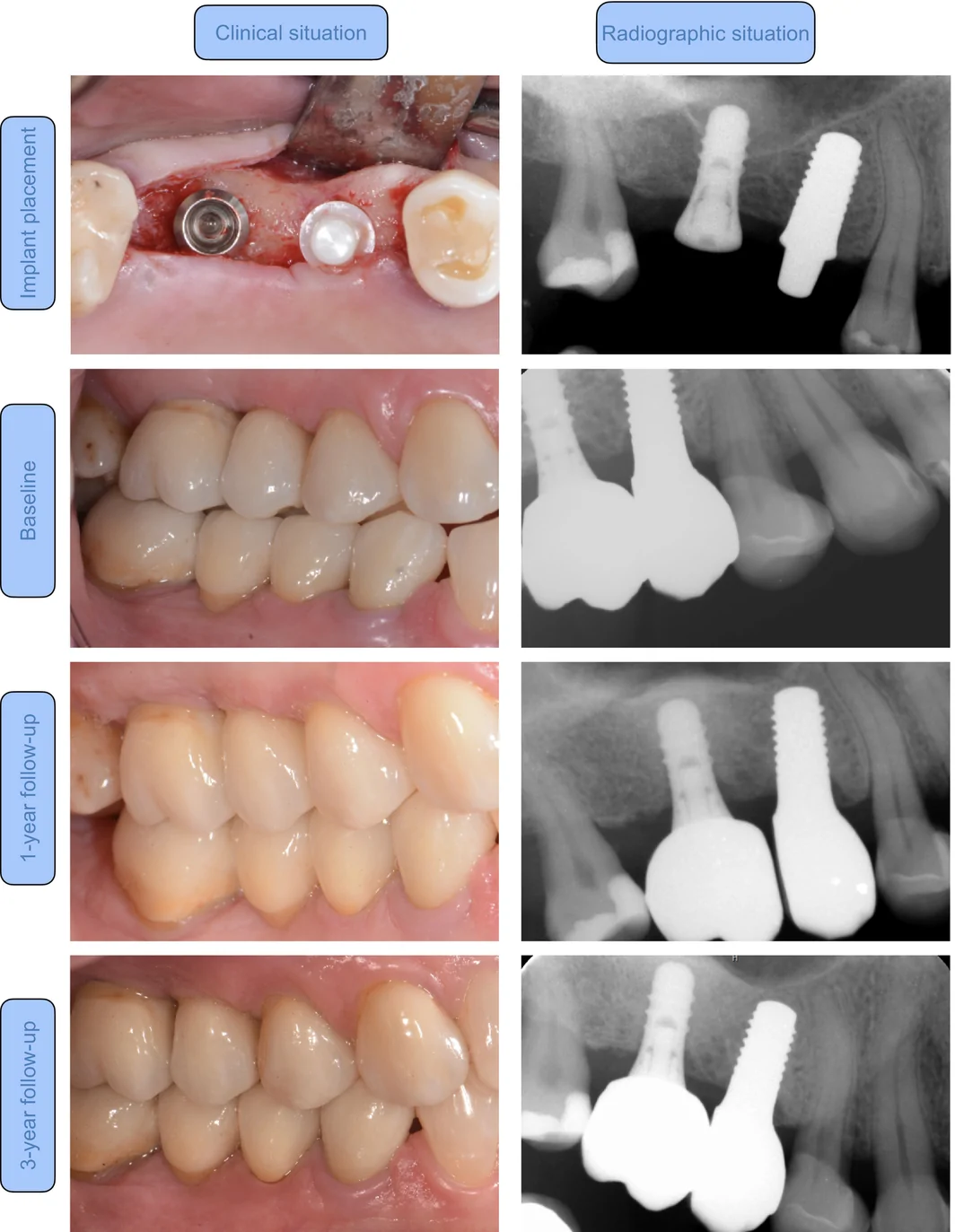
Clinical and radiographical case (implant placement, baseline, 1‐year follow‐up, 3‐year follow‐up).

### 2.4 Outcome Measures

#### 2.4.1 Clinical Parameters

At 3‐year follow‐up, the following clinical parameters were assessed by a calibrated examiner (SW) at six sites at each implant and adjacent teeth by using a periodontal probe (PCP 12; Hu Friedy, Rotterdam, The Netherlands): bleeding on probing (BOP; present/absent), plaque presence (present/absent), and probing depth (PD; mm). The width of keratinized mucosa (KT; mm) was measured with the same probe mesio‐buccal, mid‐buccal, and distal‐buccal of the implants and the neighboring teeth.

For the 3‐year follow‐up, the same criteria for peri‐implant health, mucositis criteria and peri‐implantitis were applied as in the previous publications on this cohort (Zuercher et al. 2024), following the definitions established by the 2017 World Workshop (Berglundh et al. 2018; Araujo and Lindhe 2018; Heitz‐Mayfield and Salvi 2018; Schwarz et al. 2018). Peri‐implantitis was defined by the presence of bleeding and/or suppuration on probing, an increase in probing depth compared with baseline, and progressive radiographic bone loss beyond initial physiological remodeling. To account for the subcrestal implant placement and expected initial remodeling, bone loss was considered pathological only when it extended apically beyond the rough–polished transition.

#### 2.4.2 Radiographic Measurements

Standardized intraoral radiographs were taken using the parallel technique and analyzed in ImageJ (ImageJ 1.50i, National Institutes of Health, Bethesda, MD, USA). Calibration was performed using the known thread distances of the titanium (1.25 mm) and zirconia implants (0.8 mm). Marginal bone level (MBL) was measured from the implant shoulder to the first bone‐to‐implant contact, and the position relative to the rough–smooth transition was calculated by subtracting the 1.8 mm polished collar. All radiographic measurements were performed by a single experienced and calibrated examiner (SW) using a standardized measurement protocol. In cases of uncertainty regarding the identification of the first bone‐to‐implant contact, a second experienced examiner (MB) independently assessed the radiograph, and the final measurement was established by consensus. For this study, 3‐year MBL values and changes relative to baseline and 1 year were recorded, with increases indicating bone loss and decreases indicating bone gain.

#### 2.4.3 Technical Parameters

Technical outcomes were assessed using the modified USPHS criteria. At the 3‐year follow‐up, parameters such as marginal adaptation, anatomical form, proximal contacts, occlusion, wear, color, radiographic cement gap, and chipping of the monolithic zirconia crown were re‐evaluated and graded from Alpha to Delta. Alpha indicated an optimal condition, Bravo a clinically acceptable deviation, Charlie a clinically unacceptable condition requiring intervention, and Delta a failure requiring replacement. Detailed information on the modified USPHS criteria is provided in Supporting Information (Table S1). Additional technical events, including crown retention loss at control zirconia implants and screw loosening at test titanium implants, were also recorded.

#### 2.4.4 Patient‐Reported Outcome Measures

At the 3‐year follow‐up, patient‐perceived soft‐tissue color was assessed using standardized buccal photographs and a Visual Analogue Scale (VAS). Photographs were taken with a Nikon Z6 camera and twin flash over a dental mirror and shown to patients for evaluation. Both patients and the examiner rated the color difference between the mesial and distal implant sites on a VAS from 0 (no difference) to 100 (maximum difference) and indicated a preferred site when a difference was perceived.

#### 2.4.5 Color Assessment of the Peri‐Implant Mucosa

Peri‐implant mucosal color was assessed at the 3‐year follow‐up using standardized clinical photographs. Images were analyzed by the same examiner in Adobe Photoshop. An elliptical region of interest, centered 1 mm below the mucosal margin, was selected over the peri‐implant soft tissue and over the corresponding area at the neighboring natural tooth. Lab color values were obtained, and Δ*E* values were calculated to quantify the color difference, with higher values indicating greater contrast between the compared sites.

### 2.5 Statistical Analysis

The power analysis (*n* = 40) was based on the histologically evaluated number of inflammatory cells at the level of the junctional epithelium. Data from clinical, histological, and volumetric analyses were collected via REDcap, exported to Microsoft Excel, and analyzed by using SAS software (SAS Corp., Cary, NC, USA). By applying descriptive statistics, demographic and clinical parameters, such as age, gender, smoking status, jaw, study site, and augmentation were summarized for further interpretation purposes.

Continuous numerical (marginal bone levels and changes, probing depth, keratinized mucosa, VAS scales) and implant‐level proportions of sites with plaque presence and bleeding on probing were reported as means, standard deviations, medians, and quartiles. USPHS scores were presented as categorical variables. These variables were compared over time and between treatments using generalized mixed models. Multiple comparisons within a mixed model were Bonferroni corrected. Dependent data were analyzed with McNemar test or with linear mixed models. No overall correction for multiple testing was applied to secondary endpoints. Implant‐related outcomes were analyzed at the implant level. To account for the dependency between the two implants within the same patient inherent to the split‐mouth design, patient‐level clustering was incorporated into the mixed‐effects models. The within‐patient covariance was modeled using a heterogeneous compound symmetry covariance structure or, where feasible, an unstructured covariance structure.

## 3 Results

### 3.1 Study Population/Patient Demographics

A total of 42 patients were initially enrolled, each receiving one titanium and one zirconia implant (84 implants). As reported previously (Bienz et al. 2021; Zuercher et al. 2024), two patients were excluded before baseline: one due to a medical condition preventing further treatment after implant placement and one because the biopsy specimen was of insufficient quality, resulting in 40 evaluable patients (80 implants) at baseline and the 1‐year follow‐up. At the 3‐year follow‐up, one patient was lost to follow‐up because the patient could no longer be contacted. In addition, two zirconia implants were lost (Figure 2).

**FIGURE 2:**
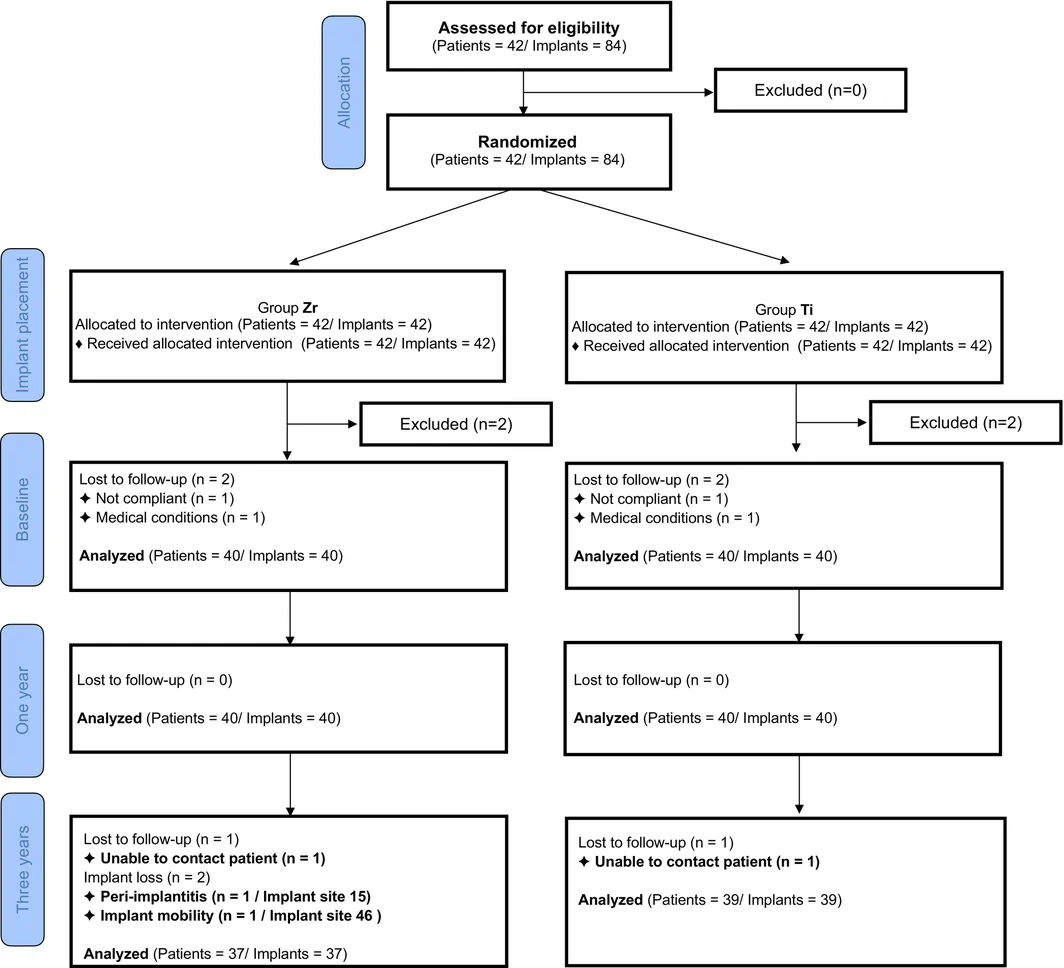
CONSORT flow diagram (*n*, number; Ti, titanium; Zr, zirconia).

### 3.2 Implant Survival

A total of 39 titanium (Ti) and 37 zirconia (Zr) implants were available for analysis, with two zirconia implants lost during the study period. One Zr implant placed at site 15 in a smoking male subject (non‐cleaning group during early mucositis simulation phase) developed peri‐implantitis and was removed 18 months after placement due to extensive, progressive bone loss. In contrast, the Ti implant did not show any marginal bone loss. The second Zr implant, placed at site 46 in a former smoker (cleaning group) with no augmentation, showed physiological clinical and radiographic parameters at follow‐up visits but became mobile and was removed 30 months after placement. The Ti implant in the same patient exhibits a bone level slightly below the transition line between the rough and smooth surface of the titanium implant. One patient was lost to the 3‐year follow‐up.

Accordingly, the 3‐year survival rate was 94.9% for Zr and 100% for Ti implants, with no significant difference between groups (McNemar test, *p* > 0.05; limited discordant pairs).

### 3.3 Clinical Outcomes

The clinical outcomes of the parameters BOP, plaque presence, PD, and KT at the time points baseline, 1, and 3 years are presented in Table 1. At the 3‐year follow‐up, the mean Bleeding on Probing (BOP) score was 29.76% (SD 26.97; 95% CI (20.77, 38.75)) in the zirconia group and 27.38% (21.45; (20.43, 34.34)) in the titanium group. At the 3‐year follow‐up, the mean percentage of peri‐implant sites with plaque was 7.7% (15.55; (2.52, 12.89)) in the zirconia group and 11.15% (17.25; (5.56, 16.75)) in the titanium group. At each time point, no statistically significant difference between zirconia and titanium implants was observed for both BOP and plaque presence.

**TABLE 1:** Clinical and radiographical outcomes at baseline, 1 year post‐loading, and 3 years post‐loading.

Variable	Treatment	Time	*N*	Mean	SD	Min	Q1	Median	Q3	Max
MBL (mm)	Zr	Post‐operative (PO)	37	−0.92	0.64	−2.05	−1.53	−0.90	−0.33	0.15
	Ti		38	−1.21	0.59	−2.65	−1.61	−1.20	−0.79	0.00
	Zr	Baseline (BL)	37	0.05	0.67	−1.75	−0.35	0.25	0.45	1.20
	Ti		39	0.09	0.80	−1.80	−0.40	0.20	0.55	1.85
	Zr	1 year	37	0.01	0.61	−1.55	−0.25	0.05	0.43	1.35
	Ti		38	0.28	0.86	−1.80	0.07	0.30	0.60	3.65
	Zr	3 years	37	0.03	0.87	−1.52	−0.44	−0.06	0.36	2.73
	Ti		38	0.24	0.61	−1.40	−0.18	0.26	0.55	1.88
		Differences								
	Zr	BL from PO	37	0.97	0.59	−0.15	0.58	1.00	1.25	2.60
	Ti		38	1.28	0.76	0.00	0.90	1.18	1.73	2.85
	Zr	1 year from PO	37	0.93	0.64	−0.40	0.45	0.80	1.40	2.90
	Ti		37	1.49	0.86	0.00	1.03	1.40	1.80	4.80
	Zr	3 years from PO	37	0.95	0.99	−0.40	0.23	0.64	1.44	3.79
	Ti		37	1.44	0.58	0.25	1.08	1.40	1.70	2.83
	Zr	1 year from BL	37	−0.05	0.40	−0.70	−0.40	−0.10	0.20	0.90
	Ti		38	0.19	0.64	−0.85	−0.16	0.10	0.41	2.65
	Zr	3 years from BL	37	−0.03	0.88	−1.47	−0.60	−0.22	0.32	2.79
	Ti		38	0.15	0.58	−0.92	−0.27	0.04	0.44	1.43
	Zr	3 years from 1 year	37	0.02	0.80	−1.12	−0.37	−0.21	0.12	3.44
	Ti		37	−0.05	0.61	−2.41	−0.33	−0.10	0.22	1.47
BOP (%)	Zr	Baseline	37	22.07	18.86	0.0	0.0	16.7	33.3	66.7
	Ti		39	14.53	18.80	0.0	0.0	0.0	33.3	66.7
	Zr	1 year	37	32.88	30.30	0.0	16.7	16.7	50.0	100.0
	Ti		39	26.07	21.90	0.0	0.0	33.3	33.3	83.3
	Zr	3 years	37	29.76	26.97	0.0	0.0	33.0	50.0	100.0
	Ti		39	27.38	21.45	0.0	16.7	33.0	50.0	66.7
Plaque presence (%)	Zr	Baseline	37	6.31	9.09	0.0	0.0	0.0	16.7	33.3
	Ti		39	6.92	12.60	0.0	0.0	0.0	16.7	50.0
	Zr	1 years	37	5.41	11.82	0.0	0.0	0.0	0.0	50.0
	Ti		39	7.26	13.68	0.0	0.0	0.0	16.7	50.0
	Zr	3 years	37	7.70	15.55	0.0	0.0	0.0	17.0	67.0
	Ti		39	11.15	17.25	0.0	0.0	0.0	17.0	67.0
PD (mm)	Zr	Baseline	37	3.00	0.55	2.0	2.5	3.2	3.3	4.2
	Ti		39	2.82	0.51	1.5	2.5	2.8	3.2	4.3
	Zr	1 year	37	2.85	0.61	1.8	2.5	2.8	3.2	5.2
	Ti		39	2.86	0.97	1.0	2.5	2.8	3.2	7.0
	Zr	3 years	37	2.97	0.78	1.5	2.5	3.0	3.3	5.3
	Ti		39	2.97	0.78	1.7	2.3	3.0	3.3	4.3
KT (mm)	Zr	Baseline	35	2.73	1.58	0.0	2.0	2.0	4.0	6.0
	Ti		37	2.74	1.26	0.0	2.0	3.0	4.0	5.0
	Zr	1 year	35	2.46	1.24	0.0	2.0	2.0	3.0	5.0
	Ti		37	2.51	1.22	0.0	2.0	3.0	3.0	5.0
	Zr	3 year	35	3.11	1.53	0.0	2.0	3.0	4.0	7.0
	Ti		37	3.30	1.56	0.0	2.0	3.0	4.0	7.0

Abbreviations: BOP, bleeding on probing; KT, keratinized tissue; MBL, marginal bone level; Min/Max, minimum; maximum; *N*, numbers; PD, probing depth; Q, quartile; SD, standard deviation; Ti, titanium; Zr, zirconia.

The mean probing depth (PD) after 3 years was 3.0 mm (0.8; (2.71, 3.23)) for the Zr group and 3.0 mm (0.8; (2.72, 3.23)) mm for the Ti group showing similar values at baseline (Zr: 3.0 ± 0.6 mm/Ti: 2.8 ± 0.6 mm) and after 1 year (Zr: 2.8 ± 0.6 mm/Ti: 2.8 ± 1.0 mm). Mean keratinized tissue width (KT) after 3 years was 3.1 mm (1.5; (2.59, 3.64)) in the zirconia group and 3.3 mm (1.6; (2.78, 3.82)) in the titanium group, with no significant difference between the groups (*p* = 0.553).

At the 3‐year follow‐up, among the 76 implants assessed, peri‐implantitis was identified in 7.9% of the implants (six implants: three titanium and three zirconia implants). Only one patient exhibited peri‐implantitis in both adjacent implants, whereas in all other cases, only a single implant was affected, with no preference for the mesial or distal position. Additionally, two zirconia implants were lost. No signs of mucositis were recorded at 18 implants (9 zirconia and 9 titanium) with no bleeding and probing at any site. In total 58 out of the 76 implants showed bleeding at least at one site when probed gently, representing 76.3% of all included implants.

### 3.4 Marginal Bone Levels and Changes

MBL measured postoperatively, at baseline, and at 1‐ and 3‐year follow‐up visits, as well as the corresponding changes over time, are presented in Table 1. Postoperatively, the mean position of the rough‐smooth interface in the Zr group was positioned 0.9 ± 0.6 mm subcrestally, whereas in the Ti group it was located at a mean of 1.2 ± 0.6 mm subcrestally (Table 1).

Between the postoperative measurement and baseline, mean MBL decreased by 1.0 ± 0.6 mm in the zirconia group and by 1.3 ± 0.8 mm in the titanium group. In both groups, the rough–smooth interface was located at a mean of 0.1 mm (Zr: 0.1 ± 0.7 mm, Ti: 0.1 ± 0.8 mm) supracrestally at baseline.

From baseline to the 3‐year follow‐up, mean MBL exhibited no change (0.0 ± 0.9 mm) for the Zr group, resulting in a rough–smooth interface located exactly at the crest (0.0 ± 0.9 mm). In contrast, the titanium group experienced an additional mean marginal bone loss of 0.1 ± 0.6 mm, positioning the bone level 0.2 ± 0.6 mm below the rough–smooth interface. Marginal bone loss between implant placement and baseline was significant in both groups (*p* < 0.001), whereas bone level changes after baseline were not statistically significant in both groups (*p* > 0.20). Significant differences of mean marginal bone levels were observed between the two groups at 1‐year follow‐up (*p* = 0.005), at 3‐year follow‐up (*p* = 0.042), whereas no significant difference was found at baseline (*p* = 0.83). No significant inter‐group differences were observed for the changes from baseline to 3 years (*p* = 0.192) or from 1 to 3 years (*p* = 0.689) (Table 2).

**TABLE 2:** Marginal bone level changes (mm) between different timepoints.

Timepoint	Implant	*N* (implants)	Mean (mm)	SD (mm)	Inter‐group *p* value^a^
Post‐operative to baseline	Ti	39	−1.3	0.8	0.008
Post‐operative to baseline	Zr	40	−1.0	0.6	
Post‐operative to 1‐year follow‐up	Ti	38	−1.5	0.9	< 0.001
Post‐operative to 1‐year follow‐up	Zr	39	−1.0	0.7	
Post‐operative to 3‐year follow‐up	Ti	37	−1.4	0.6	0.002
Post‐operative to 3‐year follow‐up	Zr	37	−0.9	1.0	
Baseline to 1‐year follow‐up	Ti	39	−0.2	0.6	0.004
Baseline to 1‐year follow‐up	Zr	39	0.1	0.4	
Baseline to 3‐year follow‐up	Ti	38	−0.1	0.6	0.192
Baseline to 3‐year follow‐up	Zr	37	0.0	0.9	
1‐year follow‐up to 3‐year follow‐up	Ti	37	0.0	0.6	0.689
1‐year follow‐up to 3‐year follow‐up	Zr	37	0.0	0.8	

Abbreviations: *N*, numbers; SD, standard deviation; Ti, titanium; Zr, zirconia.

^a^ Mixed model analysis.

### 3.5 Technical Complications

At the 3‐year follow‐up, the USPHS evaluation revealed no statistically significant differences among the various assessment criteria, with the exception of chipping. Already at baseline, one case of chipping (USPHS beta score: localized chipping, manageable by polishing) was documented in the zirconia group, while no such events were observed in the titanium group. Between baseline and the 1‐year follow‐up, the incidence of chipping (USPHS beta score) increased to two in the titanium group and to seven in the zirconia group. However, this difference did not reach statistical significance at that time (*p* = 0.100).

Between the 1‐ and 3‐year follow‐up, the number of chipping events in the zirconia group increased further, reaching a total of 10 (USPHS beta score), whereas no additional chipping was recorded in the titanium group. Consequently, at the 3‐year follow‐up, the zirconia group demonstrated a statistically significantly higher number of chipping compared to the titanium group (*p* = 0.0039). No Charlie‐rated chipping (extensive or multiple chipping not manageable by polishing) or Delta‐rated fracture requiring crown replacement occurred in either group. All USPHS scores (at baseline, 1‐ and 3‐year follow‐up) are shown in Table 3.

**TABLE 3:** USPHS: technical complications at baseline, 1, and 3 years post‐loading.

Year	Treatment group	*N* (implants)	Abutment fracture	Anatomic form	Color	Marginal adaptation	Occlusal wear	Occlusion	Patient satisfaction	Proximal contact distal	Proximal contact mesial	Veneer fracture
Baseline	Ti	39	39/0/0/0	36/3/0/0	25/14/0/0	38/1/0/0	38/1/0/0	36/1/2/0	37/2/0/0	34/2/0/0	36/2/0/0	39/0/0/0
	Zr	37	37/0/0/0	32/5/0/0	25/12/0/0	33/4/0/0	36/1/0/0	34/2/1/0	36/1/0/0	29/2/0/0	36/1/0/0	36/1/0/0
1‐year follow‐up	Ti	39	39/0/0/0	35/4/0/0	26/13/0/0	39/0/0/0	28/11/0/0	38/0/0/0	37/2/0/0	29/3/0/0	35/2/2/0	37/1/1/0
	Zr	37	37/0/0/0	31/6/0/0	25/12/0/0	35/2/0/0	26/11/0/0	35/1/0/0	35/2/0/0	25/3/1/0	31/5/1/0	30/7/0/0
3‐year follow‐up	Ti	39	39/0/0/0	33/6/0/0	26/13/0/0	37/2/0/0	20/19/0/0	36/1/1/0	37/2/0/0	24/6/2/0	27/11/1/0	36/1/0/0
	Zr	37	37/0/0/0	31/6/0/0	26/11/0/0	35/2/0/0	21/16/0/0	35/1/0/0	35/2/0/0	21/6/1/0	25/10/2/0	25/10/0/0
	Implant *p* value		1	0.259	0.888	a	0.813	a	a	0.21	0.925	0.001
	Time *p* value		1	0.421	0.866	a	< 0.001	a	a	0.04	< 0.001	0.027

*Note:* Rating: Alpha/Beta/Charlie/Delta; Chisq *p*: test the time and implant effect, interaction was never significant; a: no solution for the model.

Abbreviations: *N*, numbers; Ti, titanium; Zr, zirconi.

Over the 3‐year observation period, two cases of decementation were recorded in the zirconia group. The first event occurred after 17 months of functional loading and the second after 11 months. Both were successfully managed through recementation. In the titanium group, two instances of screw loosening were observed: one at 20 months and the second at 5 months post‐loading.

### 3.6 Patient‐Reported Outcomes

At follow‐up appointments, both patients and the examiner evaluated the color of the peri‐implant soft tissues using mirrored buccal photographs and a Visual Analogue Scale (VAS). The results at the 1‐year follow‐up have been reported previously. The VAS scores reported by patients at the 3 years follow‐up were 20.56 ± 25.11. Similarly, the examiner's ratings showed 19.80 ± 24.08 in the mean VAS scores. There was no statistically significant difference between the evaluations provided by the professional examiner and those of the lay patients (0.76 ± 18.68, *p* = 0.841).

When the cohort was stratified by gender, female patients reported greater perceived differences in mucosal color (mean VAS: 25.67 ± 29.98) compared to male patients (mean VAS: 15.73 ± 18.99). This trend was mirrored in the assessments provided by the (female) examiner, who also recorded higher VAS scores for female participants (females: 27.94 ± 30.02; males: 12.08 ± 13.41). Although both patient‐ and examiner‐reported VAS scores were consistently higher in female participants, the observed gender‐related differences failed to reach statistical significance. The differences of the mean VAS scores of patients and examiners were not significant for both genders (*p* > 0.34).

In cases where a color difference was perceived by patients (*n* = 26), there was a tendency to prefer the soft tissue coloration around the zirconia implant site. At the 3‐year follow‐up, 15 patients (57.7%) expressed a preference for the mucosa adjacent to the zirconia implant, while 11 patients (42.3%) favored the titanium site.

The professional examiner identified a perceptible color difference in 31 cases. Among these, the examiner preferred the zirconia site in 20 instances (65.5%) and the titanium site in 11 instances (35.5%).

### 3.7 Color Assessment of the Peri‐Implant Mucosa

The Δ*E* values (color differences, all are positive) at all time points between titanium and zirconia implants, to the adjacent tooth, are presented in Table 4. At the 3‐year follow‐up, the Δ*E* values relative to the neighboring tooth were 13.54 (SD 7.25; 95% CI (11.49, 16.00)) for zirconia implants and 13.75 (6.66; 11.49, 16.00) for titanium implants. The mean ΔE difference between zirconia and titanium implants was 0.21 (1.08; (−1.99, 2.41)).

**TABLE 4:** Color assessment of the peri‐implant mucosa between baseline, 1‐ and 3‐year post‐loading.

	Treatment	Mean	SD	Min	Q1	Median	Q3	Max
Prior surgery								
Delta‐E value implant to neighboring tooth	Zr	13.49	6.69	3.32	8.20	12.80	16.86	32.40
	Ti	14.29	6.29	4.58	10,83	14.35	18.32	30.25
Baseline								
Delta‐E value implant to neighboring tooth	Zr	11.63	5.62	1	7.87	10.84	13.81	26.25
	Ti	11.63	5.96	1	8.23	10.93	15.43	26.48
1‐year follow‐up								
Delta‐E value implant to neighboring tooth	Zr	10.10	5.28	1.41	6.56	8.66	13.42	26.17
	Ti	11.08	4.07	2.83	8.06	10.49	14.21	18.47
3‐year follow‐up								
Delta‐E value implant to neighboring tooth	Zr	13.54	7.25	3.61	7.38	11.54	18.62	30.68
	Ti	13.75	6.66	4.36	8.35	13.17	18.49	32.20

Abbreviations: Min/Max, minimum, maximum; *N*, numbers; Q, quartile; SD, standard deviation; Ti, titanium; Zr, zirconia.

The mean Δ*E* of the implant materials and time did not show a significant interaction term (mixed model *p* = 0.777). Without interaction, the mixed model showed no implant significant effect (*p* = 0.338). Only the time effect was significant (*p* = 0.008); in particular, the mean Δ*E* prior to surgery and the 1‐year follow‐up and prior to surgery and the 3‐year follow‐up were significant (*p* = 0.048 resp. *p* = 0.009, Bonferroni corrected).

## 4 Discussion

The present randomized, controlled clinical within‐subject study assessed the clinical performance of zirconia and titanium implants placed in posterior sites at the 3‐year follow‐up. The findings showed that (1) both implant types demonstrated stable peri‐implant health, (2) with statistically significant but clinically no relevant differences in marginal bone levels, and (3) esthetic and patient‐reported outcomes were similar. However, zirconia implants exhibited a slightly lower survival rate and a higher incidence of chipping, indicating material‐specific considerations that remain clinically relevant.

The 3‐year survival rate was 100% for titanium and 94.9% for zirconia implants. Both zirconia implant failures occurred after the first year—one in a smoker developing peri‐implantitis and one presenting with late mobility despite previously healthy peri‐implant tissues (cleaning group). Although slightly lower, the zirconia survival rate did not differ statistically from titanium. These findings are consistent with clinical studies reporting similarly high zirconia implant survival, typically ranging from 91% to 98% at 3–5 years and approximately 92%–95% at up to 10 years (Balmer et al. 2020; Gahlert et al. 2016; Kohal et al. 2023). A slightly lower survival rate of zirconia implants in the present study may also relate to characteristics of the monotype design and the intraoral cementation procedure. This observation is further supported by a recently published randomized controlled trial using the same titanium implants as a control group but a two‐piece screw‐retained zirconia implant design, which demonstrated a higher survival rate for the ceramic implants. Overall, the present data reinforce that zirconia implants can achieve comparable early survival yet may carry a slightly higher risk of mid‐term complications or failure (Balmer, Payer, et al. 2026).

Radiographically, the remaining implants demonstrated stable marginal bone levels over the 3‐year period. After the initial remodeling phase, bone levels remained largely unchanged, with zirconia implants maintaining the rough–smooth interface at the crestal level and titanium implants showing only minimal additional bone loss. Although differences at 1 and 3 years were statistically significant, the small magnitude indicates limited clinical relevance. Furthermore, bone remodeling is strongly influenced by prosthetic load transfer rather than implant material alone, and the standardized restorative approach applied in the present study likely contributed to similar outcomes in both groups (Jung et al. 2017). These observations agree with previous reports documenting minimal bone loss around both implant materials when placed and restored according to established protocols (Balmer, Payer, et al. 2026; Gahlert et al. 2016; Kohal et al. 2020).

Clinical peri‐implant soft‐tissue parameters remained stable and comparable between zirconia and titanium implants. No statistically significant differences were observed for BOP, plaque presence, probing depth, or keratinized tissue width. Although zirconia surfaces have been shown in preclinical studies to accumulate less plaque and result in a lower inflammatory response (Kohal et al. 2004; Scarano et al. 2004), the trend toward lower plaque accumulation around zirconia implants observed in the present study did not translate into lower bleeding scores. One explanation may be that crowns on zirconia implants were cemented intraorally, introducing a small risk of undetectable residual cement—a known contributor to soft‐tissue inflammation (Linkevicius et al. 2013; Wilson Jr. 2009). In contrast, titanium implant crowns were cemented extraorally on a titanium base and screw‐retained, minimizing cement‐related irritation. Therefore, the biological outcomes should be interpreted with caution, as the potential effects of implant material cannot be clearly separated from those of the different prosthetic retention protocols. Nevertheless, the incidence of peri‐implantitis at 3 years was low and did not differ between both groups, aligning with systematic reviews reporting no clinically relevant differences in soft‐tissue health between zirconia and titanium implants (Padhye et al. 2023).

In contrast to the biological outcomes, technical complications revealed distinct material‐related differences. Chipping of the monolithic crowns occurred significantly more frequently in the zirconia implant group and increased progressively toward the 3‐year follow‐up. This pattern agrees with previous reports on monotype (one‐piece) zirconia implants, where ceramic chipping is the most common prosthetic complication, particularly in posterior regions (Balmer, Spies, et al. 2026; Hashim et al. 2016; Spitznagel et al. 2022). Potential contributing factors include the brittle nature of zirconia under flexural loading. Furthermore, the one‐piece implant design may result in a more direct transfer of occlusal forces, as it lacks an intermediate abutment interface that could help dissipate functional stress (Spies et al. 2016). Titanium implants, in contrast, showed two episodes of screw loosening—a well‐known and usually minor complication associated with two‐piece systems (Goodacre et al. 2003)–and none of these events compromised implant survival.

Esthetic outcomes and patient‐reported satisfaction were comparable between the two implant systems. Δ*E* values relative to neighboring teeth showed no significant differences, and both patients and the examiner‐reported low levels of perceived color discrepancy. When differences were noticed, there was a modest preference for the zirconia implant sites, consistent with earlier findings indicating reduced mucosal discoloration over zirconia (Thoma et al. 2016) and with the 1‐year outcomes from the same cohort (Zuercher et al. 2024). However, these differences were small and did not translate into meaningful differences in overall satisfaction. Thus, while zirconia implants may offer an esthetic advantage, this effect was measured in posterior regions where soft‐tissue visibility is low.

Strengths of this study include its randomized split‐mouth design, which minimizes inter‐patient variability, and the uniform surgical and prosthetic protocols, including the use of monolithic zirconia implant crowns for both implant types. Limitations include the moderate sample size, the exclusive focus on posterior regions, and the use of a monotype zirconia implant design, which may not fully reflect the performance of newer two‐piece zirconia systems now commonly used in clinical practice. Furthermore, the study was originally powered for the histological primary endpoint following the experimental mucositis phase rather than for comparisons of long‐term outcomes between titanium and zirconia implants. Therefore, comparisons between the two implant materials should be interpreted with appropriate caution. The absence of formal examiner reproducibility testing and the highly standardized single‐center setting should also be considered when interpreting the findings.

Within these limitations, the 3‐year findings indicate that zirconia implants perform comparably to titanium implants with respect to peri‐implant health, bone stability, esthetic outcomes, and patient satisfaction. Nevertheless, zirconia implants showed a slightly lower survival rate and a significantly higher incidence of chipping, highlighting the need for material‐specific considerations when selecting implants for posterior regions. Further research with larger cohorts, longer observation periods, and two‐piece zirconia implant systems is warranted to better understand long‐term performance and technical reliability.

## 5 Conclusion

The present study demonstrated stable peri‐implant health and comparable marginal bone levels for both zirconia and titanium implants over 3 years. Patient‐reported outcomes and esthetic measures were similar between the two systems. However, zirconia implants showed a slightly lower survival rate and a higher incidence of chipping. Overall, both implant systems performed similarly in posterior sites during the mid‐term 3 follow‐up.

## Author Contributions

**Marc Balmer:** investigation, data curation, formal analysis, project administration, writing – original draft, writing – review and editing. **Ronald E. Jung:** conceptualization, investigation, writing – review and editing, funding acquisition. **Anina N. Zuercher:** investigation, writing – original draft, writing – review and editing, formal analysis, data curation. **Stephanie A. Wenk:** data curation, formal analysis, writing – original draft. **Daniel S. Thoma:** conceptualization, investigation, funding acquisition, writing – review and editing. **Stefan P. Bienz:** investigation, data curation, formal analysis, writing – review and editing.

## Funding

The study is funded by a grant of the International Team for Implantology (1018‐2014). Material support was granted by Straumann Holding AG.

## Conflicts of Interest

M.B., S.P.B., D.T., S.A.W., and A.N.Z. report grants from ITI, Straumann, VITA, Prosec, Osteology, and Geistlich during the conduct of the study. R.E.J. reports grants, personal fees, and other support from Geistlich, Straumann, VITA, Henry Schein, ITI, Osteology, and TRI during the conduct of the study.

## Supporting information


**Table S1:** Modified United States Public Health Service (USPHS) criteria applied for the assessment of technical outcomes. A, Alpha; B, Bravo; C, Charlie; D, Delta.

**Data S1:** CONSORT 2025 checklist of information to include when reporting a randomized trial*.

## Data Availability

The data that support the findings of this study are available on request from the corresponding author. The data are not publicly available due to privacy or ethical restrictions.
